# Experimental proof of Joule heating-induced switched-back regions in OLEDs

**DOI:** 10.1038/s41377-019-0236-9

**Published:** 2020-01-10

**Authors:** Anton Kirch, Axel Fischer, Matthias Liero, Jürgen Fuhrmann, Annegret Glitzky, Sebastian Reineke

**Affiliations:** 1 Dresden Integrated Center for Applied Physics and Photonic Materials (IAPP) and Institute of Applied Physics, Technische Universität Dresden, Nöthnitzer Straße 61, 01187 Dresden, Germany; 2 Weierstrass Institute Berlin, Mohrenstraße 39, 10117 Berlin, Germany

**Keywords:** Electronics, photonics and device physics, Organic LEDs

## Abstract

Organic light-emitting diodes (OLEDs) have become a major pixel technology in the display sector, with products spanning the entire range of current panel sizes. The ability to freely scale the active area to large and random surfaces paired with flexible substrates provides additional application scenarios for OLEDs in the general lighting, automotive, and signage sectors. These applications require higher brightness and, thus, current density operation compared to the specifications needed for general displays. As extended transparent electrodes pose a significant ohmic resistance, OLEDs suffering from Joule self-heating exhibit spatial inhomogeneities in electrical potential, current density, and hence luminance. In this article, we provide experimental proof of the theoretical prediction that OLEDs will display regions of decreasing luminance with increasing driving current. With a two-dimensional OLED model, we can conclude that these regions are switched back locally in voltage as well as current due to insufficient lateral thermal coupling. Experimentally, we demonstrate this effect in lab-scale devices and derive that it becomes more severe with increasing pixel size, which implies its significance for large-area, high-brightness use cases of OLEDs. Equally, these non-linear switching effects cannot be ignored with respect to the long-term operation and stability of OLEDs; in particular, they might be important for the understanding of sudden-death scenarios.

## Lighting: Modelling the electrothermal performance of organic light-emitting diodes

Modelling the impact of electrically induced heat on the performance of organic light-emitting diodes (OLEDs) could pave the way for their use in large-area lighting applications, including general lighting, signage and automobiles. In recent years, OLEDs have become ubiquitous in lighting and display technology because of their high power efficiency and sharp colours. However, their luminance diminishes with the increased current density required for high-brightness large-area lighting applications, an effect that is related to Joule self-heating. Now, Sebastian Reineke and colleagues from the Technische Universität Dresden and Weierstrass Institute Berlin in Germany have, for the first time, demonstrated so-called ‘switched-back’ regions of decreased luminance on a lab-sized OLED display. By modelling the electrothermal characteristics of OLEDs, the researchers could lay the foundations for handling these effects in large-area lighting applications, such as car lights and flexible lighting panels.

## Introduction

In recent decades, non-thermal artificial light sources have replaced their thermal counterparts in almost all areas. Light-emitting diodes (LEDs) have become the most applied lighting and display technology because they feature high power efficiency and sharp colours. While cheap and easy-to-process planar devices, such as organic LEDs (OLEDs)^1,2^, hold an evolving market share for displays^3^, major manufacturers are just on the edge of implementing them in large-area lighting scenarios, e.g., as car rear lights or flexible lighting panels^4,5^. These possible applications require high-brightness operation, for which the OLED technology does not yet meet the industry specifications. Thus, OLEDs are not competitive in these large-scale markets^6–8^. Studying high-current phenomena and understanding non-linear switching effects, which can lead to abrupt failure of OLEDs and may compromise their performance, is essential to the future development of this technology. Such investigations, however, require reliable electrothermal models incorporating a thermally activated electrical conductivity that may lead to non-linear behaviour.

**Fig. 1:**
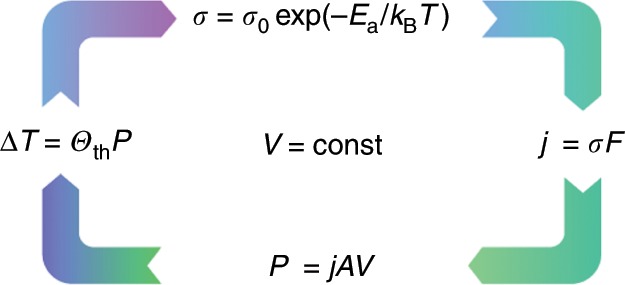
Feedback loop of electrothermal coupling. The current density *j* causes power dissipation *P*, raising the sample temperature *T* according to the thermal resistance *Θ*_th_ and, consequently, electrical conductivity *σ*. In the case of a constant applied voltage *V*, this leads to an increase in the current density and hence a thermal runaway situation.

Lab-size OLEDs are well capable of providing the high luminance levels exceeding 10,000 cd/m^2^ needed for lighting use^9^. When extending the device architecture to a large area, however, several issues require further performance optimisation. Finding transparent electrodes with low sheet resistance that allow for two-dimensional scaling, for instance, is still an important research topic^10,11^. Current solutions, such as using thin films of indium tin oxide (ITO), lead to inhomogeneous voltage conditions throughout the device area^12^. In connection with Joule self-heating and electrothermal feedback (cf. Fig. 1 and refs. ^13,14^), this results in substantial spatial brightness variations^15–17^. Improved lateral heat dissipation may pose a possible answer to this problem. However, it is important that such advances provide compatibility with state-of-the-art substrate solutions^18–20^.

Recent studies in the area of OLED self-heating revealed that these devices can feature bistable behaviour and show S-shaped current–voltage characteristics, which are well known from thermistor theory^21–23^. It has been shown that Joule heating and electrothermal feedback locally cause a regime of negative differential resistance (NDR, increasing current density at decreasing voltage)^24^. Based on a comprehensive model, it was predicted that this effect will eventually cause parts of the emission zone to be switched back in luminance (SB, decreasing local voltage AND current density) while the total device supply current still grows^25^. To date, the observation of this effect has not been reported.

Here, we experimentally prove the existence of SB regions by taking camera images under increasing driving current. After an explanation of the effect, we develop a model that can well reproduce the measured data for two different test cases. Finally, the model is used to predict the device behaviour for large areas. The experiments shown here not only prove the existence of such unprecedented effects but also demonstrate the validity of comprehensive electrothermal modelling in general that can be the basis for the understanding and optimisation of any thin-film, large-area LED technology, where other examples are LEDs based on quantum dots or perovskites.

## Results

### Experimental setup

All experiments are carried out using a red, top-emitting standard OLED with a p-doped/intrinsic/n-doped (p-i-n) layer stack fabricated by thermal evaporation of small molecules under vacuum. The 20 nm thick emission layer comprises Spiro-TAD doped with 10% of the red phosphorescent emitter Ir(MDQ)_2_(acac), as widely discussed in the literature^26,27^ (see Methods and SI for further details). This architecture is chosen to achieve a temperature-stable device, which can be stressed with high-current densities before degradation. Two nanometres of gold and 7 nm of silver are used as a transparent top contact (*R*_S_ = 15 Ω/sq).

**Fig. 2:**
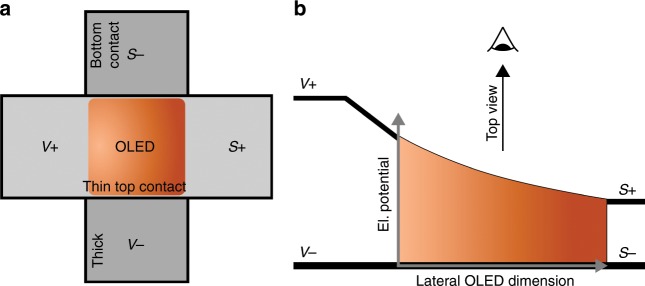
**a** Top-view sketch of the 4-wire setup. The bottom electrode consists of 80 nm of aluminium and silver, while the transparent top contact is made from 2 nm of gold and 7 nm of silver. Its significant sheet resistance leads, as indicated, to a left-to-right luminance gradient across the pixel. **b** V+ refers to the supplied and S+ refers to the sensed potential. Due to the thick bottom electrode, the contacts V- and S- can be assumed to be on the same potential (ground).

To exclude the impact of electrical resistances outside the active area, we implement a 4-wire crossbar measurement (cf. Fig. 2a). Herein, two electrodes (V+ and V−) are used as the current supply (*J* = *J*_sup_), while another pair of contacts (S+ and S−) is applied for sensing only (*J* = 0) in a crossbar structure^25^. In this configuration, the current can be taken as running from the left top contact (V+) to the grounded bottom electrode (ϕ_V−_ = ϕ_S−_). The qualitative electrical potential distribution within the OLED sample is plotted in Fig. 2b. It outlines the influence of the contact resistance, which induces the applied potential drop before even reaching the active area, and of *R*_electrode_ on the luminance homogeneity.

**Fig. 3:**
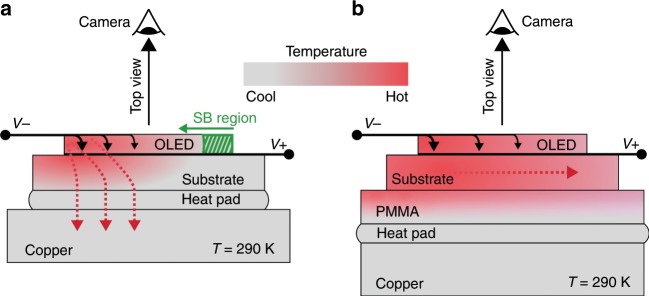
Qualitative heat flow chart of the sample arrangements realised in our experiments. One OLED is placed directly onto a copper support (**a**) and another equal device onto a PMMA sandwich layer (**b**). The heat flux is indicated by red and the electric current by black arrows.

To ensure isothermal surroundings, all measurements are carried out in a Peltier cryostat, where the device rests on a temperature-controlled (*T* = 290 K) copper support. A thin heat-conducting soft pad introduced as a sandwich layer between the copper support and substrate glass (cf. Fig. 3) smooths out potential interface inhomogeneities and accounts for the decent vertical thermal.

To further screen our measurements from unwanted external influences, all data are taken under vacuum to prevent natural convection^28^ and water condensation. The luminance intensity of the OLED is logged by a top-mounted USB camera (Basler acA1920-40uc, Basler AG, Germany) using the control software SweepMe!^29^. To ensure both simultaneous image recording from above and temperature stabilisation from below, a top-emitting OLED is used.

Two experimental scenarios are realised for measuring the current–voltage characteristics of our samples. In the first scenario, the device is placed directly onto the copper/heat pad (cf. Fig. 3a). In the second, as indicated in Fig. 3b, an additional layer of poly(methyl methacrylate) (PMMA) is sandwiched between the substrate and heat pad.

### Experimental findings

**Fig. 4:**
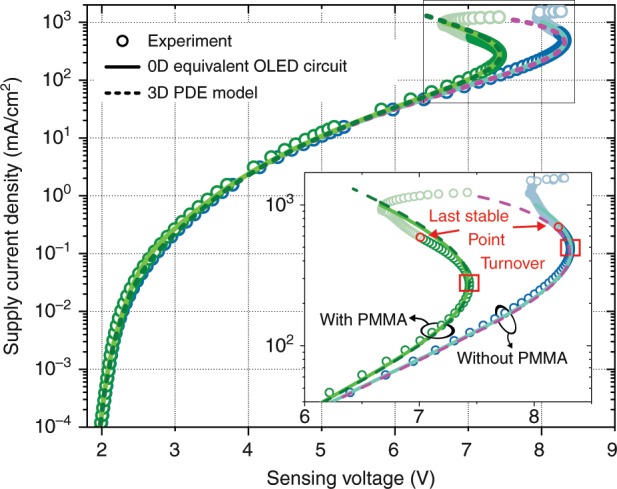
Current–voltage curves for both OLED arrangements in Fig. 3. The inset is a zoomed-in view of the S-bend, where an elevated current density (and hence electrical conductivity) can be observed for the device with PMMA (green circles) with respect to the case without PMMA (blue circles). Both the turnover and last stable points are indicated. Model data are discussed later in this section.

Figure 4 presents the current-stabilised current–voltage characteristics for both experimental scenarios (cf. Fig. 3) using two equal OLEDs. Each data point is presented as a circle. At every current step, the sample rests for 2 s to ensure thermal equilibrium throughout the device before taking a voltage measurement. Meanwhile, the luminance of the OLED pixels is tracked by taking one camera image for each current step.

Both OLED samples pass through the Shockley and charge transport-limited regimes before showing their distinctive S-shapes at current densities above 100 mA/cm^2^. The inset of Fig. 4 is a zoomed-in view of the region of interest. The turnover point represents the condition at which the total differential resistance in the samples vanishes. Due to the temperature-stable device architecture, it is possible to further stress the OLEDs deep into their NDR region, where the occurrence of switching effects is expected.

Apparently, the device with an additional PMMA layer shows a turnover point at lower current density and voltage. As PMMA is a poor heat conductor, functioning as a significant vertical heat barrier that prevents heat from leaving the device, the OLED is expected to experience a more pronounced temperature increase with respect to the device placed directly onto the copper support and driven with the same supply current (cf. SI Figs. S2 and S3). Hence, its electrical conductivity rises more rapidly, which explains its shifted current–voltage curve.

**Fig. 5:**
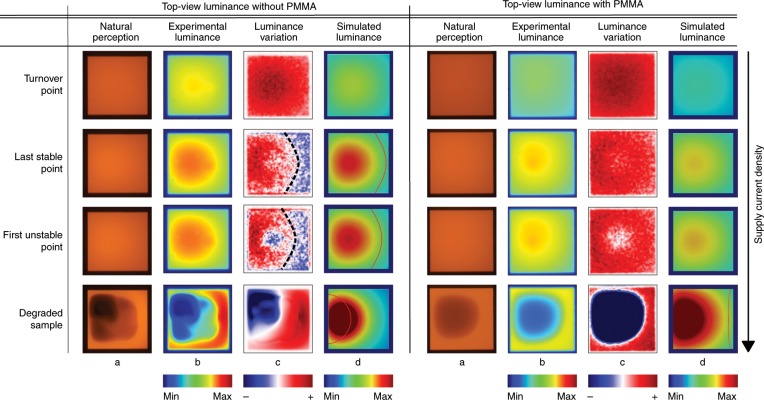
Distinct images taken with a camera while running current-stabilised scans for both settings (with and without the PMMA layer). Column **a** shows the OLED pixel as it appears to the human eye (natural perception), while the images in **b** have a recalculated colour coding representing the actual luminance. To emphasise the device’s luminance evolution, column **c** plots the difference relative to the picture taken in the previous current density step. The experimental data are compared to simulated luminance results from the PDE model at respective points of the current–voltage scan, as shown in **d**. The corresponding current density points are indicated in Fig. 4. The OLED stack without PMMA shows a luminance SB region before degradation, which can also be found by simulation (SB boundaries indicated by a red line). Introducing a PMMA sandwich layer postpones the occurrence of an SB region beyond the sample's degradation point (cf. SI videos).

Figure 5 presents the camera pictures taken at the respective points indicated in Fig. 4. Column a shows the OLED area as it appears to the naked eye. These pictures are recalculated to absolute luminance and plotted in a different colour coding in column b. Frame c depicts the luminance variation with respect to t previous data point. It thus indicates how the brightness of the OLED changes with increasing supply current and identifies regions of increasing (red) and decreasing (blue) luminance.

The OLED placed directly onto the copper support shows regions of switched-back luminance above the turnover point. The boundary of its migrating SB region is indicated by a black dashed line. By contrast, the sample with the additional PMMA layer does not feature any such behaviour.

Stressing the devices even further will eventually result in their degeneration. Degraded areas of the device exhibit abruptly increased electrical resistance and thus a plummeting luminance. This can be most clearly identified from column c when an image shows a blue spot emerging from the active area's centre (as can be followed in the SI videos). The last measurement point before a degeneration spot occurs is called the last stable point, and all subsequent data points are greyed out in colour (cf. Fig. 4). With the help of the camera images, the SB effect can be well separated from degradation. As shown in Fig. 5c, the shapes of the SB and degradation regions are significantly different. Device instabilities start to evolve from a small spot in the centre of the active area, whereas SB regions are found to migrate into the pixel from the right side (far from V+).

### Understanding switched-back regions

These interesting experimental results arise from non-linear, thermally induced behaviour that requires a more in-depth understanding of the governing physical principles.

**Fig. 6:**
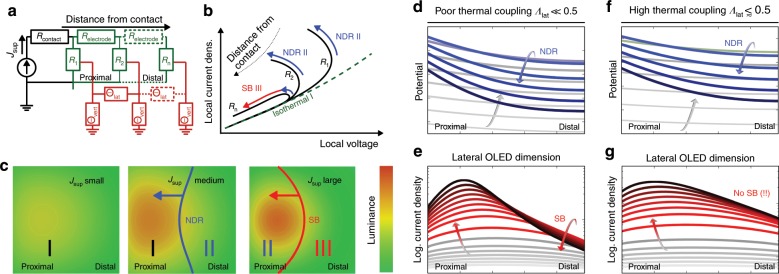
**a** Sketch of a thermistor array for understanding the S-NDR and switching effects in an OLED stack. **b** Indication of local JV characteristics depending on the distance from the electrode. **c** Top-view of OLED pixel luminance with increasing supply current density *J*_sup_. The PDR (I), NDR (II) and switched-back luminance (III) regions are indicated. Potential and current cross-sections of a device **d**, **e** with poor and **f**, **g** with enhanced lateral thermal coupling, respectively. Frames **c**–**g** originate from the 3D numerical model with Eq. (4) used throughout our work and demonstrate NDR and SB regions migrating into the pixel from the right (distal) side.

The SB effect can be explained by treating the OLED as a 1D array of thermistors^25,30^, as introduced in Fig. 6a. The device comprises a transparent top electrode with significant sheet resistance *R*_electrode_ and a highly conductive thick bottom electrode. The current is supplied from the top-left side and runs to the bottom electrode of the device. With the OLED modelled as an array of thermistors (electrical wiring drawn in green), each self-heating thermistor is represented by its own self-consistent S-shaped current–voltage curve. The point of operation on each curve is, however, influenced by each thermistor’s position with respect to the supply electrode. Distal thermistors experience lower voltages and local currents due to *R*_electrode_ (*J*_k_ > *J*_k+1_). Concerning the thermal conditions (indicated by red lines), each thermistor *R*_k_ with temperature *T*_k_ is connected to an infinite heat sink via a thermal resistance *Θ*_vert_, which is realised in the experiment by the copper support being constantly kept at *T*_ref_ = 290 K. Additionally, every thermistor is thermally connected to its adjacent neighbours via a lateral thermal resistance *Θ*_lat_. Within the framework of Fourier's heat conduction law, the lateral thermal coupling

1 $${\mathrm{\varLambda }}_{{\mathrm{lat}}} = \frac{{{\mathrm{\varTheta }}_{{\mathrm{vert}}}}}{{{\mathrm{\varTheta }}_{{\mathrm{lat}}} + 2{\mathrm{\varTheta }}_{{\mathrm{vert}}}}},0 \le {\mathrm{\varLambda }}_{{\mathrm{lat}}} \le \frac{1}{2}$$

can be defined, which describes Δ*T*_k_ = *Λ*_lat_(*T*_k+1_, *T*_k−1_, *T*_ref_) as being dependent on the temperature of its adjacent elements with respect to *T*_ref_ (see SI for further details). Due to the symmetry of the system, *Λ*_lat_ is assumed to be constant throughout the entire device except for at its edges. In the following, two cases that correspond to the experimentally applied scenarios are discussed.

In the first case, the *Θ*_vert_ for every thermistor *R*_k_ is assumed to be small (*Λ*_lat_ ≪ 0.5). Upon running a current-stabilised current–voltage scan, every thermistor experiences increases in voltage, current density, heat dissipation and, consequently, conductivity and luminance (isothermal regime I, positive differential resistance (PDR), cf. Fig. 6b). However, with a sufficiently high *R*_electrode_, *R*_1_ is well ahead of *R*_n_ in terms of the local current density, and hence, its conductivity increase must be more pronounced, which implies a more rapid decrease in the differential resistance (DR). *T*_n_ can be imagined to be much smaller than *T*_1_ and thus *R*_n_ less conductive than *R*_1_. At some point, the DR of *R*_1_ will be small enough to prevent *V*_n_ from rising any further. If the supply current continues to increase, then *V*_n_ will even start to fall, and as a result, *R*_n_ experiences an NDR (regime II). The NDR region enters the device from the right side (far from the supply electrode) and migrates towards the left, as indicated in Fig. 6c providing a top-view of an OLED pixel. The scenario of locally falling potentials with increasing supply current is outlined in Fig. 6d. The behaviour of *R*_n_ when stressing the device even further depends on its distance to the supply electrode (as indicated in Fig. 6b). If the distance is sufficiently large (*n* · *R*_electrode_ ≫ *R*_n_), then the thermistor will only note a decrease in voltage caused by the NDR of more proximal thermistors. When *R*_n_ is cold enough, it immediately jumps back into a PDR regime (falling current AND voltage) and returns to its initial curve, potentially showing a certain hysteresis. This is defined as a regime of switched-back (SB) current (III) and can be related to a decreasing local current density (and hence luminance) with rising total current (cf. Fig. 6e). The greater the decrease in the resistance of proximal thermistors is, the greater the relative significance of *R*_electrode_. This also causes regime III to migrate left in the direction of the supply electrode, as exemplarily outlined in Fig. 6c.

In the second case, the vertical thermal resistance *Θ*_vert_ for every thermistor *R*_k_ is considered to be high (*Λ*_lat_ ⪅ 0.5). In the experiment, this is realised by adding a PMMA sandwich layer that accounts for an additional vertical heat barrier between the OLED and copper support. As the thermistor array can now be assumed to be closer to thermal equilibrium, the conductivity deviations between individual thermistors are reduced. As a consequence, the SB region only enters the device at higher currents, which are no longer experimentally accessible with our setup. Fig. 6d–g show actual data from an implemented 3D heat and current simulation, which is subsequently introduced. They indicate the cross-sectional distributions of the local potential and current density with increasing supply current, depending on *Λ*_lat_. The changes in line colour from grey to red/blue indicate the entrance of an NDR region into the device. For the current densities realised in our experiments, the simulation yields an SB region in the case of *Λ*_lat_ ≪ 0.5, whereas none is achieved for *Λ*_lat_ ⪅ 0.5.

Figure 6c exemplarily translates the cross-sectional data into the top-view appearance of an OLED pixel and emphasises the consequences of non-linear electrothermal coupling (regions I and II rising luminance, region III falling luminance).

To quantitatively compare the understanding of the electrothermal effects with our experimental results, two different model approaches are realised, which are introduced in the following sections.

### Electrothermal 0D OLED equivalent circuit

**Fig. 7:**
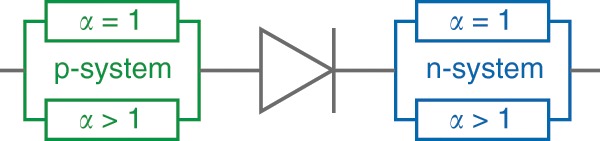
Charge carrier recombination represented by the ideal diode equation. The charge transport of holes (p-system) and electrons (n-system) is modelled by the temperature-activated conductivity of linear and non-linear resistors.

Recently, a full electrothermal OLED model that includes non-linear self-heating effects was presented^30^. This approach is implemented here using LTSpice to reproduce the entire current–voltage curve of the OLED, including the Shockley, charge transport-limited, and self-heating regimes. It comprises a simple stack of a p-doped system, an intrinsic recombination zone, and an n-doped system (Fig. 7).

Charge carrier recombination, which is assumed to occur predominantly in the emission zone, is described by the recombination current of the ideal diode equation:

2 $$j_{{\mathrm{rec}}}\left( {V,T} \right) = j_{00}{\mathrm{exp}}\left( {\frac{{\mathrm{e}V - E_{a,0}}}{{n_{{\mathrm{id}}}k_{\mathrm{B}}T}}} \right)$$

The migration of charge carriers on distinct energy levels separated by *E*_a,0_ is assumed. The ideality factor *n*_id_ takes non-ideal behaviour into account and typically ranges between 1 and 2. The maximum possible recombination current is described by *j*_00_, the externally applied voltage by *V* and the thermal energy by *k*_B_*T*.

Both the p- and n-systems are represented by a parallel connection of the linear (α = 1) and super-linear (α > 1) temperature-activated current–voltage relation:

3 $$j\left( {V,T} \right) = j_{{\mathrm{ref}}}\left( {\frac{V}{{V_{{\mathrm{ref}}}}}} \right)^\alpha {\mathrm{exp}}\left[ {\frac{{ - E_{\mathrm{a}}}}{{k_{\mathrm{B}}}}\left( {\frac{1}{T} - \frac{1}{{T_{{\mathrm{ref}}}}}} \right)} \right]$$

with arbitrarily chosen *T*_ref_ = 290 K and *V*_ref_ = 1 V, at which the current is measured to be *j*_ref_. Further details of the model can be found in ref. ^30^.

The dependence on thermal coupling in the experiment is implemented with a tuneable global thermal resistance that connects all the abovementioned parts of the thermistor stack to an infinite heat reservoir (*T* = 290 K) representing the copper support in the experiment. Approximately the same fitting parameters are used as previously reported for the same device architecture (cf. Table 1 and ref. ^30^).

**Table 1:** Electrical parameters for simulation.

Model	Parameter	Value	
Recombination	*j*_00_	40 A/cm²	
	*E*_a,0_	2.74 eV	
	*n*_id_	1.55	
p-system		*Linear*	*Non-linear*
	*α*	1.0	4.1
	*j*_ref_	0.6 mA/cm²	8.1 mA/cm²
	*E*_a,0_	0.275 eV	0.25 eV
n-system		*Linear*	*Non-linear*
	*α*	1.0	3.07
	*j*_ref_	0.05 mA/cm²	3.3 mA/cm²
	*E*_a,0_	0.5 eV	0.325 eV

Electrical parameters used for both the 0D electrothermal OLED simulation and 3D PDE model to match the experimental data. These values were taken from a previous study in our lab where the same OLED architecture was used and were only slightly modified^30^

As the 0D model successfully matches the experimental data using solely its tuneable global thermal resistance (*Θ*_without PMMA_ = 115 K/W and *Θ*_with PMMA_ = 225 K/W) to switch between both experimental scenarios (cf. Fig. 4), the same set of parameters is taken for a further 3D simulation to yield temperature and current distributions.

### 3D PDE model of heat and current flows

Herein, we study the stationary heat and current flows for the two different setups (cf. Fig. 3 a, b). In the first case, only the OLED, the glass substrate, and the heat pad between the glass substrate and copper support are considered. In the second case, we additionally include the PMMA layer between the glass substrate and the heat pad. We compute a 3D numerical solution of the following partial differential equations (PDEs):

4 $$\begin{array}{*{20}{c}} { - \nabla \cdot \left( {{\it{\upsigma \nabla \varphi }}} \right) = 0,} \\ { - \nabla \cdot \left( {\lambda \nabla T} \right) = \sigma \left| {{\it{\nabla }}\varphi } \right|^2} \end{array}$$

where the first equation describes the current flow driven by the potential *φ* through the OLED device and is solved only in the subdomain comprising the OLED stack itself. The conductivity function *σ* = *σ*(*x*, *T*, |∇*φ*|) incorporates the dependence on temperature *T* and electric field strength |∇*φ*| as in the zero-dimensional case (Eqs. (2), (3)). More details on the model can be found in ref. ^31^. The electrical parameters that enter the conductivity function *σ* are collected in Table 1. The second formula in Eq. (4) is the heat equation for temperature *T* with the power dissipation density due to Joule heating. The thermal parameters of the simulation are listed in Table 2. From the solutions of the PDE system in Eq. (4), the current density *j* = *σ*∇*φ* and, in turn, the luminance *L* = *L*(*j*) can be computed (see Methods for further details).

**Table 2:** Additional dimensional and thermal parameters used for the PDE model.

Device	Parameter	Value
OLED	Lateral dimensions	2.54 mm × 2.54 mm
Glass substrate	Thermal conductivity	1.2 W/(m K)^42^
	Lateral dimensions	2.54 cm × 2.54 cm
	Thickness	1.1 mm
Heat pad	Thermal conductivity	0.48 W/(m K)^a^
	Thickness	0.5 mm
PMMA	Thermal conductivity	0.21 W/(m K)
	Lateral dimensions	2.54 cm × 2.54 cm
	Thickness	2 mm
Copper support	Thermal conductivity	300 W/(m K)^42^

Private conversation with company: the thermal conductivity given in the data sheet is only to be considered at pressures that we cannot reach with our setup. Furthermore, the vertical thermal coupling is potentially compromised by the non-perfect interfaces

^a^Softtherm pad 86/600, Kerafol, data sheet: 6 W/(m K)

The glass substrate is given by [0, *L*] × [0, *H*_gls_] × [0, *W*], where the length and width are chosen sufficiently large such that boundary conditions have no influence. The thickness of the glass substrate is *H*_gls_ = 1.1 mm. The additional PMMA layer has the same length and width, but its thickness is *H*_PMMA_ = 2.0 mm. The heat pad has thickness *H*_pad_ = 0.5 mm, and the copper support is realised by Dirichlet boundary conditions using *T*_cop_ = 290 K.

Note that due to the high electrical conductance of the bottom electrode, it is replaced by Dirichlet boundary conditions at the bottom of the organic layers. Moreover, due to symmetry, only half of the structure is simulated. The influence of the atmosphere in the encapsulation glass is marginal since most of the heat is dissipated into the copper block. Hence, the atmosphere is not included in the simulation, and Robin boundary conditions are used instead.

For the numerical solution of Eq. (4), we implement a code based on the toolbox *pdelib*^32^ developed and maintained at WIAS. This code uses spatial discretization based on a Voronoi box finite volume scheme and an iterative strategy using the sparse matrix direct solver *PARDISO*^33^.

## Discussion

Figure 5 clearly demonstrates the appearance of SB regions in the OLED pixel with poor lateral thermal coupling *Λ*_lat_ that rests directly on the copper support (without PMMA) at current densities above the turnover point. A comparison of the experimental luminance data and the PDE model shows convincing agreement, i.e., the simulation reproduces the appearance and even the shape of SB regions for the respective current densities.

In the case of an increased *Λ*_lat_ (with PMMA), we can show that the model-based prediction of the appearance of switching effects is shifted to higher current densities, hence these effects are not appearing in the investigated device characteristics before degradation.

**Fig. 8:**
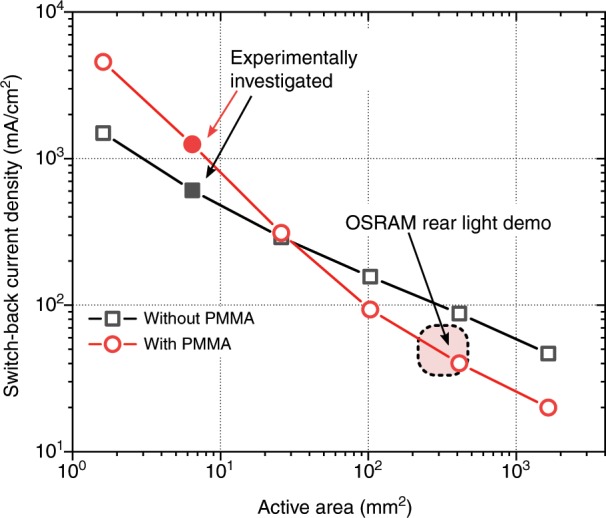
The PDE model with Eq. (4) allows for extending the experimentally accessible range towards large-pixel areas. Keeping all other parameters unaltered, it can be demonstrated that SB regions start to enter the device at a lower current density (switch-back current) with increasing pixel size. Parameters approximated from an OSRAM rear light demonstrator are indicated for comparison (see SI for details)^38–41^.

As both scenarios of thermal coupling (with and without PMMA) can be modelled by the same set of parameters, the PDE model provides the possibility of extending our experimentally accessible range of active device areas. Figure 8 shows the correlation between pixel size and *j*_SB_, the supply current density at which SB occurs for the first time within the OLED emission area. This calculation shows that *j*_SB_ decreases with pixel size. Since we have already demonstrated SB regions in lab-scale devices (*A* = 6.45 mm^2^), we expect them to become of major significance when applying high currents to extended large-area planar light-emitting devices. The estimation predicts that the currents used in OLED car rear lights might already be sufficient to cause SB effects. Furthermore, considering, e.g., a 10 cm × 10 cm OLED panel, the estimation suggests that the electrothermal treatment is not exclusively relevant to high-brightness and lighting applications but possibly relevant even to conditions below 1000 cd/m^2^.

Surprisingly, Fig. 8 suggests that enhanced lateral thermal coupling (with PMMA) does not simply scale up *j*_SB_ for extended active areas, as found in this work. In contrast, for OLED dimensions exceeding lab size, poor *Λ*_lat_ is expected to reduce the vulnerability of devices to switching effects, as the significance of the sheet resistance of the transparent electrode increases drastically. The interplay between the thermal coupling, area size, and non-linear effects must be considered when scaling up OLEDs for high-brightness applications, as unexpected physical phenomena may arise.

The demonstration of SB phenomena by the PDE model also proves their origin to be electrothermal in nature. Additionally, the regional appearance of SB regions (migrating in from the right for the given geometry) and degradation (starting in the central spot) observed in the camera images in Fig. 5c proves that SB is not related to degradation.

In summary, we have used a lab-sized OLED as an example to demonstrate the non-linear electrothermal behaviour of thin-film light-emitting devices and proved the occurrence of switched-back regions under high-brightness conditions. Regardless of the increasing total current, these OLED regions experience decreasing potential drop, local current density, and thus luminance. This effect is induced by inhomogeneous Joule heating, whose gradual nature is caused by the significant ohmic resistance of the transparent electrode and insufficient lateral thermal coupling. Using a 0D equivalent circuit and a 3D PDE model, we have demonstrated the tremendous importance of correct electrothermal, non-linear modelling of thin-film LEDs under high-brightness operation. We could successfully reproduce the entire range of experimental data (Shockley, charge transport-limited, NDR and SB regimes) using the same set of fitting parameters. Additionally, the geometrical appearance of SB regions could be reproduced by the PDE model. The modelling further allowed us to study the scaling of the above non-linearities, suggesting that large-area devices will experience an increasing impact of the SB effect. Furthermore, lateral decoupling of heat exchange was found to promote the encountered switch-back effect for the investigated devices. This information is of major importance when long-term stability is addressed, as an efficient OLED cooling system may become an ambiguous measure. This decoupling shifts the turnover point to higher current densities and prevents thermally induced degradation. On the other hand, it may render the device more vulnerable regarding switched-back regions and may lead to catastrophic failure of large-area devices driven at the high currents needed for future, high-brightness lighting applications. Our findings can be extended from OLEDs to similar thin-film LED architectures that show temperature-activated conductivity, such as quantum dot or perovskite LEDs.

## Materials and methods

### OLED fabrication

OLEDs were fabricated by thermal evaporation under high vacuum (Kurt J. Lesker Company) on 1 inch-by-1 inch glass substrates of 1.1 mm thickness. The OLEDs have the commonly used p-i-n structure. Aluminium and silver (40 nm each) are used as bottom electrodes. The p-layer consists of 40 nm of Spiro-TTB:C_60_F_36_ (4 wt%), the electron-blocking layer of 10 nm of Spiro-TAD, the i-layer of 20 nm of Spiro-TAD doped with 10 wt% Ir(MDQ)_2_(acac), the hole-blocking layer of 10 nm of BAlq_2_, and the n-layer of 65 nm of TPBi:Cs (1:1). On top of this stack, a transparent top electrode was deposited (Au 2 nm and Ag 7 nm). Please find the full names of the materials and *J*-*V*-*L* characteristics in the SI.

To prohibit air and moisture contamination, the OLED stacks were encapsulated under a nitrogen atmosphere after fabrication. The encapsulation glass features a small cavity above the OLED pixels that prevents direct contact between sensitive materials and the encapsulation glass. It was attached to the substrate using an epoxy resin (XNR5516Z-L and XNR5590, Nagase Europa GmbH).

The device architecture was chosen for its high thermal stability and was not optimised in terms of efficiency. EQE and luminance values of 10.7% and 1000 cd/m^2^ are reached at ~40 mA/cm^2^ (cf. SI Fig. S2). The brightness shortly before degradation is estimated to reach 5000 cd/m^2^ at current densities of ~700 mA/cm^2^.

### Four-wire measurement

For the four-wire measurements, a dual-channel Keithley 2602 A SMU was used and controlled by SweepMe! Software^29^. The air pressure in our cryostat was reduced to below 1 mbar using a pre-vacuum pumping system (Trivac D16B, Germany). Such a vacuum proves to be sufficient to render thermal convection insignificant in our experiments.

As a PMMA layer, a standard 10 cm × 10 cm × 2 mm acrylic glass panel was purchased and cut to the OLED glass substrate size (2.54 cm × 2.54 cm).

### Numerical 3D PDE method

For the numerical solution of the 3D thermistor system of partial differential equations (PDEs), we created a code based on the toolbox pdelib^32^ developed and maintained at WIAS. A detailed description of the model can be found in ref. ^31^ and the references therein. For visualisation of solutions, we used the open-source software ParaView. The formula used for the calculation of the luminance *L*(j) is

5 $$\begin{array}{*{20}{c}}L\left( j \right) = C \times {\mathrm{EQE}}_0 \times \frac{{j_0}}{4} \times \left( {\sqrt {1 + \frac{{8j}}{{j_0}}} - 1} \right)\end{array}$$

where *C* = 5 cd/m^2^ is a normalisation constant, *j*_0_ = 45 mA/cm^2^ is a reference current density, and EQE_0_ = 10.7% is the external quantum efficiency in the absence of triplet–triplet annihilation (comp. ref. ^34^).

The spatial discretization was based on a hybrid finite-volume/finite-element approach on tetrahedral meshes^35^. The basic discretization scheme for both the heat conduction and current conservation equations was a finite-volume method, which uses Voronoi cells constructed from the tetrahedral meshes as control volumes. The potential gradient entering the non-linear conductivity coefficient was reconstructed using a finite element ansatz on the tetrahedra of the discretization. The approach features global and local heat conservation and guarantees that the numerical solution remains within the a priori bounds of the solution of the continuous heat flow equation.

Since the lateral dimensions of the active region are 1000 times larger than its vertical dimensions (100 nm), the structure is highly anisotropic. Tensor product meshes were used for computation. Each of these meshes exactly reproduces an approximation of the device geometry by anisotropic cuboids. To obtain the final computational grids, each cuboid was further subdivided into six tetrahedra. The strong anisotropy leads to large condition numbers of the linear system of equations arising from the discretization of the PDE system. An iterative strategy based on the sparse direct solver PARDISO^33,36,37^ combined with path-following techniques ensured that the correct solution was obtained and the S-shaped current–voltage characteristics with negative differential resistance could be traced. In the path-following method, the voltage increments were automatically adjusted. In particular, smaller increments were used before voltage turnover points.

The computational grid has to be fine near the active region, whereas a coarser mesh can be used to resolve the surrounding part. For the structure without the additional layer, the mesh consisted of approximately 67,000 vertices and 360,000 tetrahedra, while the structure with PMMA had approximately 70,000 vertices and 370,000 tetrahedra.

## Supplementary information

Supplementary Material

Video 1-Experiment_with_PMMA

Video 2-Experiment_without_PMMA

Video 3-PDE_model_with_PMMA

Video 4-PDE_model_without_PMMA

## Data Availability

The data that support the findings of this study are available from the corresponding author upon reasonable request.

## References

[1] Kamtekar KT, Monkman AP, Bryce MR (2010). Recent advances in white organic light-emitting materials and devices (WOLEDs). Adv. Mater..

[2] Liang JF (2016). Recent advances in high performance solution processed WOLEDs for solid-state lighting. J. Mater. Chem..

[3] Kathirgamanathan P (2015). Electroluminescent organic and quantum dot LEDs: the state of the art. J. Disp. Technol..

[4] Bobbert P, Coehoorn R (2013). A look inside white OLEDs. Europhys. N..

[5] Mertens, R. *The OLED Handbook*. (OLED-Info, 2018).

[6] Bergemann KJ, Krasny R, Forrest SR (2012). Thermal properties of organic light-emitting diodes. Org. Electron..

[7] Spindler J (2016). High Brightness OLED Lighting. SID Symp . Dig. Tech. Pap..

[8] Nardelli A (2017). Assessment of Light Emitting Diodes technology for general lighting: a critical review. Renew. Sustain. Energy Rev..

[9] Reineke S (2009). White organic light-emitting diodes with fluorescent tube efficiency. Nature.

[10] Kim YH (2013). Achieving high efficiency and improved stability in ITO-free transparent organic light-emitting diodes with conductive polymer electrodes. Adv. Funct. Mater..

[11] Jung S (2014). Extremely flexible transparent conducting electrodes for organic devices. Adv. Energy Mater..

[12] Park J, Lee J, Noh YY (2012). Optical and thermal properties of large-area OLED lightings with metallic grids. Org. Electron..

[13] Kirsch, C. et al. Electrothermal simulation of large-area semiconductor devices. *Int. J. of Multiphysics* **11**, 127–136 (2017).

[14] Krikun G, Zojer K (2019). Impact of thermal transport parameters on the operating temperature of organic light emitting diodes. J. Appl. Phys..

[15] Gärditz C (2007). Impact of Joule heating on the brightness homogeneity of organic light emitting devices. Appl. Phys. Lett..

[16] Neyts K (2006). Inhomogeneous luminance in organic light emitting diodes related to electrode resistivity. J. Appl. Phys..

[17] Slawinski M (2013). Investigation of large-area OLED devices with various grid geometries. Org. Electron..

[18] Schwamb P, Reusch TCG, Brabec CJ (2013). Passive cooling of large-area organic light-emitting diodes. Org. Electron..

[19] Chung S (2009). Substrate thermal conductivity effect on heat dissipation and lifetime improvement of organic light-emitting diodes. Appl. Phys. Lett..

[20] Zakhidov AA (2012). Hydrofluoroethers as heat-transfer fluids for OLEDs: operational range, stability, and efficiency improvement. Org. Electron..

[21] Becker JA, Green CB, Pearson GL (1946). Properties and uses of thermistors— thermally sensitive resistors. Trans. Am. Inst. Electr. Eng..

[22] Burgess RE (1955). Fluctuations of the numbers of electrons and holes in a semiconductor. Proc. Phys. Soc. Sect. B.

[23] Popescu C (1975). The effect of local non-uniformities on thermal switching and high field behaviour of structures with chalcogenide glasses. Solid-State Electron..

[24] Fischer A (2013). Self-heating, bistability, and thermal switching in organic semiconductors. Phys. Rev. Lett..

[25] Fischer A (2014). Feel the heat: nonlinear electrothermal feedback in organic LEDs. Adv. Funct. Mater..

[26] Meerheim R (2008). Influence of charge balance and exciton distribution on efficiency and lifetime of phosphorescent organic light-emitting devices. J. Appl. Phys..

[27] Saragi TPI, Fuhrmann‐Lieker T, Salbeck J (2006). Comparison of charge-carrier transport in thin films of Spiro-linked compounds and their corresponding parent compounds. Adv. Funct. Mater..

[28] Pohl L, Kohári Z, Poppe A (2018). Vertical natural convection models and their effect on failure analysis in electro-thermal simulation of large-surface OLEDs. Microelectron. Reliab..

[29] Fischer, A. & Kaschura, F. *SweepMe!.* https://sweep-me.net (2019).

[30] Fischer A (2018). Full electrothermal OLED model including nonlinear self-heating effects. Phys. Rev. Appl..

[31] Liero M (2017). 3D electrothermal simulations of organic LEDs showing negative differential resistance. Opt. Quantum Electron..

[32] Fuhrmann, J. et al. *WIAS-Software. Software components for PDEs*. https://www.pdelib.org (2018).

[33] Schenk O, Gärtner K (2004). Solving unsymmetric sparse systems of linear equations with PARDISO. Future Gener. Comput. Syst..

[34] Baldo MA, Adachi C, Forrest SR (2000). Transient analysis of organic electrophosphorescence. II. Transient analysis of triplet-triplet annihilation. Phys. Rev. B.

[35] Fuhrmann, J., Glitzky, A. & Liero, M. Hybrid finite-volume/finite-element schemes for *p*(*x*)-laplace thermistor models. in *Finite Volumes for Complex Applications VIII—Hyperbolic, Elliptic and Parabolic Problems* (eds Cancès, C. & Omnes, P.) 397–405 (Springer, Cham, 2017).

[36] Schenk O, Gärtner K (2006). On fast factorization pivoting methods for sparse symmetric indefinite systems. Electron. Trans. Numer. Anal..

[37] Karypis G, Kumar V (1998). A fast and high quality multilevel scheme for partitioning irregular graphs. SIAM J. Sci. Comput..

[38] Hofmann S (2011). Top-emitting organic light-emitting diodes. Opt. Express.

[39] Kim KH (2014). Phosphorescent dye-based supramolecules for high-efficiency organic light-emitting diodes. Nat. Commun..

[40] Ràfols-Ribé J (2018). High-performance organic light-emitting diodes comprising ultrastable glass layers. Sci. Adv..

[41] OSRAM OLED GmbH. *Segmented OLED rearlight demonstrator, www.osram-oled.com/applications*. https://www.osram-oled.com/anwendungen/p001_anwendung_segmentierte_oled.jsp (2019).

[42] Fischer A (2012). Self-heating effects in organic semiconductor crossbar structures with small active area. Org. Electron..

