# Supplementary material for: Experimental proof of Joule heating-induced switched-back regions in OLEDs
Source: Light Sci Appl. 2020 Jan 10;9:5. doi: 10.1038/s41377-019-0236-9 (PMC6954226; doi:10.1038/s41377-019-0236-9)
Supplement: Supplementary file 1 — Supplementary Material [file 41377_2019_236_MOESM1_ESM.pdf]

Supplementary Information

Experimental proof of Joule heating-induced switched-back regions in OLEDs

Anton Kirch,[1] Axel Fischer,[1,*] Matthias Liero,[2] Jürgen Fuhrmann,[2] Annegret Glitzky,[2] and Sebastian Reineke[1]

[1]*Dresden Integrated Center for Applied Physics and Photonic Materials (IAPP) and Institute of Applied Physics, Technische Universität Dresden Nöthnitzer Straße 61, 01187 Dresden, Germany*

[2]*Weierstrass Institute Berlin, Mohrenstraße 39, 10117 Berlin, Germany*

(Dated: December 3, 2019)

* axel_fischer@tu-dresden.de

I. OLED MATERIALS AND CHARACTERISTICS

Table S1: List of organic molecules used for OLED manufacturing.

Spiro-TTB	2,2',7,7'-tetrakis(N,N'-di-p-methylphenylamino)-9,9'-spirobifluorene
Spiro-TAD	2,2',7,7'-tetrakis(diphenylamino)-9,9'-spirobifluorene
$Ir(MDQ)_2(acac)$	iridium (III) bis(2-methyldibenzo[f,h]quinoxaline) (acetylacetonate)
TPBi	2,2',2"-(1,3,5-benzinetriyl)-tris(1-phenyl-1-H-benzimidazole)
$BAlq_2$	Aluminum (III) bis(2-methyl-8- quinolinato)-4-phenylphenolate

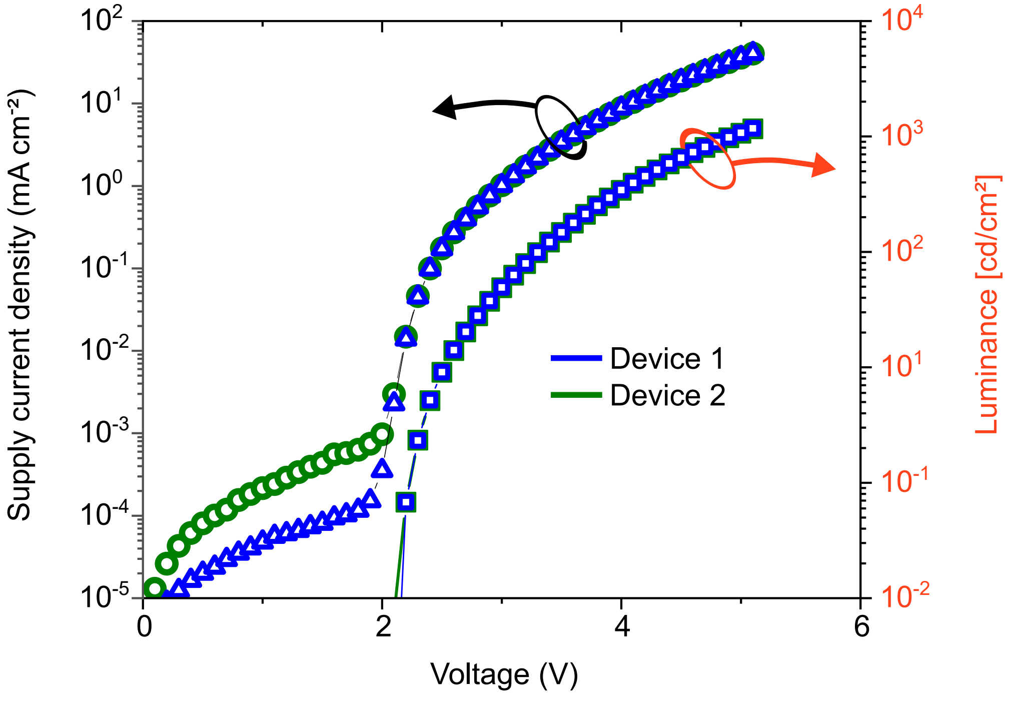

Figure S1: Luminance and current density characteristics for the two OLEDs used. The measurements were carried out in pulsed mode until 5.1 V to prevent degradation. Both devices comprise the same stack and were fabricated in the same batch. Device 1 was later used for the experiment *with PMMA* and device 2 for the experiment *without PMMA*.

II. TEMPERATURE DISTRIBUTION THROUGHOUT THE OLED DEVICE

The numerical 3D model provides access to the local temperature landscape of the devices. Figures S2 and S3 demonstrate the main difference of the two scenarios at $J_{\text{sup}} = 500\,\text{mA/cm}^2$. The main body of the pictures shows the OLED's glass substrate. In the case of Fig. S3, the PMMA layer can be made out below the glass substrate. Please note that due to the system's symmetry only half of the OLED device is considered. Also, each sample rests on a copper support that is considered as infinite heat reservoir below the sample (boundary condition $T_{\text{ref}} = 290\,\text{K}$). The small rectangle in the middle indicates the OLED pixel.

Two main deductions can be made from these images: First, while in case of *without PMMA* the glass substrate remains relatively cool and the generated heat is transfered rather vertically, the additional PMMA layer causes the heat to spread all over the substrate. Second, this leads to an elevated pixel temperature in case of *with PMMA* and also to a more homogeneous temperature distribution within the active area.

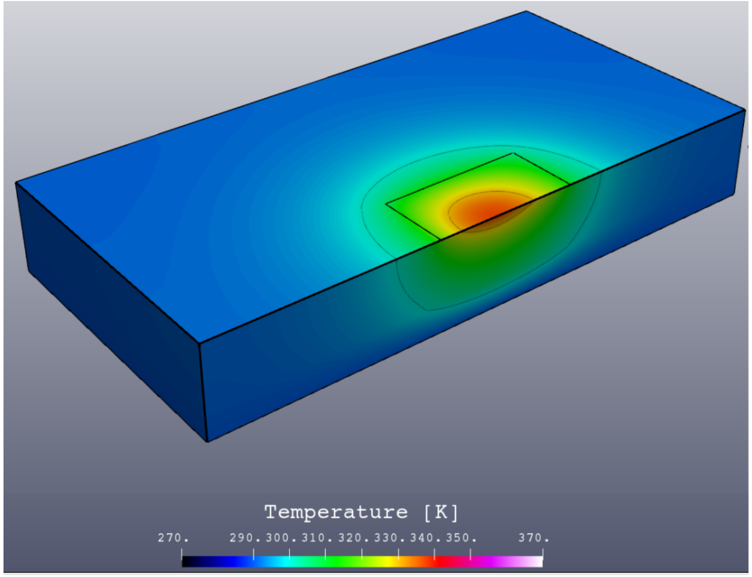

Figure S2: Temperature distribution in the OLED device (black rectangle) and glass substrate *without PMMA* at $J_{\text{sup}} = 500\,\text{mA/cm}^2$.

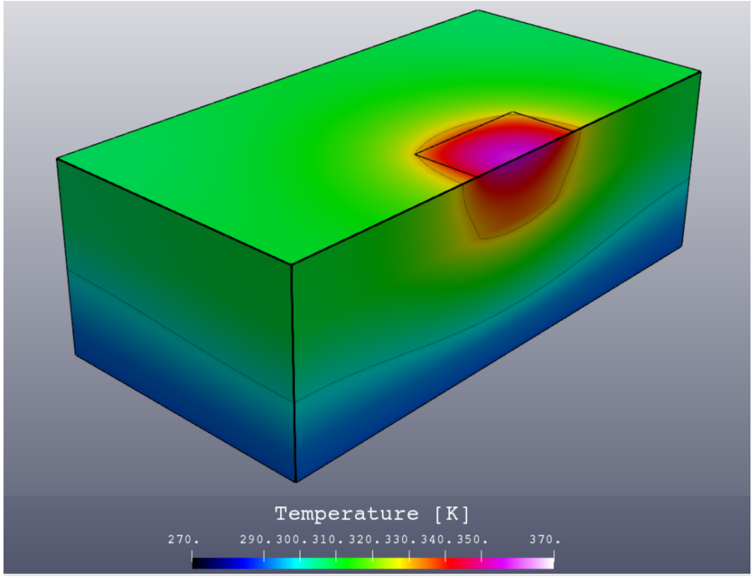

Figure S3: Temperature distribution of the device OLED (black rectangle) and glass substrate *with PMMA* at $J_{\mathrm{sup}} = 500\,\mathrm{mA/cm^2}$.

III. CURRENT DENSITIY IN OLED REAR LIGHTS

The OSRAM rear light demonstration states a total device area of $A = 100\,\mathrm{cm^2}$ including 30 OLED modules of $A = 333\,\mathrm{mm^2}$ each. For fulfilling European road traffic regulations, they report a luminance level of $L = 7500\,\mathrm{cd/m^2}$ [8]. As no further details on applied current densities could be found, we estimated them by comparing the luminance value to literature values of highly-efficient red-emitting devices and found it to be in the range of $30\,\mathrm{mA/cm^2} - 80\,\mathrm{mA/cm^2}$ [9–11].

IV. DEFINITION OF LATERAL THERMAL COUPLING

The definition of Λ_{lat} is based on Fourier's law that defines a heat flux $\dot{Q}$ to be

$$\dot{Q} = \frac{T_{\mathrm{W1}} - T_{\mathrm{W2}}}{\Theta_{\mathrm{th}}} \tag{1}$$

for the 1D case, as depicted in Fig. S4. The thermal resistance is written as Θ_{th} and T_{W} is the temperature of a certain heat reservoir. In order to determine the temperature impact onto any (except for the outermost) thermistor in the 1D array due to its nearest elements, we used the definition of

$$\Lambda_{\mathrm{lat}} = \frac{\Theta_{\mathrm{vert}}}{\Theta_{\mathrm{lat}} + 2\Theta_{\mathrm{vert}}}, \tag{2}$$

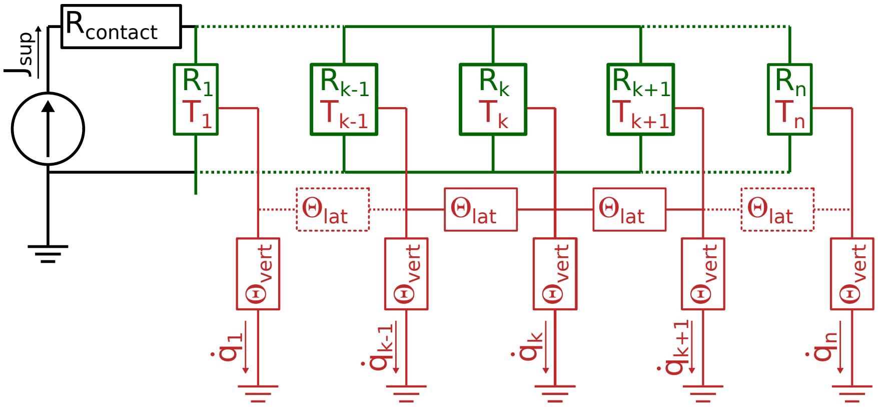

Figure S4: The definition of lateral thermal coupling Λ_{lat} can be understood by treating the OLED as 1D chain of thermistors.

as stated in the main article. Note that for this definition, we consider ΔT to be the temperature change of R_{k} solely due to the heat flux from/to adjacent thermistors and not due to self-heating! Since the definition of the thermal resistance

$$\Theta_{\text{th}} = \rho_{\text{th}} \cdot \frac{d}{A}, \tag{3}$$

with ρ_{th} being the thermal resistivity, d the length and A the area of the heat-resistive material, depends on geometrical settings, we assume a squared geometry ($A = d^2$ for both Θ_{lat} and Θ_{vert}). As Λ_{lat} is only used as phenomenological value, this assumption does not influence any calculation.

The lateral thermal coupling was derived for any thermistor R_{k} with temperature T_{k} via Fourier's law and $\dot{q}_{\text{i}} = \frac{T_{\text{i}}}{\Theta_{\text{vert}}}$ as follows.

$$\begin{aligned}
\Delta T_{\text{k}} &= T_{\text{k}-1} - \Theta_{\text{lat}} \sum_{\text{k}}^{\text{n}} \dot{q}_{\text{i}} \\
&= T_{\text{k}-1} - \Theta_{\text{lat}} \dot{q}_{\text{k}} - \Theta_{\text{lat}} \sum_{\text{k+1}}^{\text{n}} \dot{q}_{\text{i}} \\
&= T_{\text{k}-1} - \Theta_{\text{lat}} \frac{T_{\text{k}} - T_{\text{ref}}}{\Theta_{\text{vert}}} - \Theta_{\text{lat}} \sum_{\text{k+1}}^{\text{n}} \dot{q}_{\text{i}} \\
&= T_{\text{k}-1} - \frac{\Theta_{\text{lat}}}{\Theta_{\text{vert}}} (T_{\text{k}} - T_{\text{ref}}) - (T_{\text{k}} - T_{\text{k}+1}) \\
&= \underbrace{\frac{\Theta_{\text{vert}}}{2\Theta_{\text{vert}} + \Theta_{\text{lat}}}}_{\Lambda_{\text{lat}}} \left(T_{\text{k}-1} + T_{\text{k}+1} + \frac{\Theta_{\text{lat}}}{\Theta_{\text{vert}}} T_{\text{ref}} \right)
\end{aligned} \tag{4}$$

[1] Juergen Fuhrmann, T Streckenbach, H Langmach, and M Uhle, "WIAS-Software," (2018).

[2] Matthias Liero, Juergen Fuhrmann, Annegret Glitzky, Thomas Koprucki, Axel Fischer, and Sebastian Reineke, "3d electrothermal simulations of organic LEDs showing negative differential resistance," Optical and Quantum Electronics **49**, 330 (2017).

[3] M. A. Baldo, C. Adachi, and S. R. Forrest, "Transient analysis of organic electrophosphorescence. II. Transient analysis of triplet-triplet annihilation," Physical Review B **62**, 10967–10977 (2000).

[4] Jürgen Fuhrmann, Annegret Glitzky, and Matthias Liero, "Hybrid Finite-Volume/Finite-Element Schemes for p(x)-Laplace Thermistor Models," in *Finite Volumes for Complex Applications VIII - Hyperbolic, Elliptic and Parabolic Problems*, Springer Proceedings in Mathematics & Statistics, edited by Clément Cancès and Pascal Omnes (Springer International Publishing, 2017) pp. 397–405.

[5] Olaf Schenk and Klaus Gärtner, "Solving unsymmetric sparse systems of linear equations with PARDISO," Future Generation Computer Systems Selected numerical algorithms, **20**, 475–487 (2004).

[6] Olaf Schenk and Klaus Gärtner, "On fast factorization pivoting methods for symmetric indefinite systems," Electronic Transactions on Numerical Analysis **23**, 158–179 (2006).

[7] G. Karypis and V. Kumar, "A Fast and High Quality Multilevel Scheme for Partitioning Irregular Graphs," SIAM Journal on Scientific Computing **20**, 359–392 (1998).

[8] "OSRAM OLED GmbH, Segmented OLED rearlight demonstrator, www.osram-oled.com/applications," (2019).

[9] Simone Hofmann, Michael Thomschke, Björn Lüssem, and Karl Leo, "Top-emitting organic light-emitting diodes," Optics Express **19**, A1250–A1264 (2011).

[10] Kwon-Hyeon Kim, Sunghun Lee, Chang-Ki Moon, Sei-Yong Kim, Young-Seo Park, Jeong-Hwan Lee, Jin Woo Lee, June Huh, Youngmin You, and Jang-Joo Kim, "Phosphorescent dye-based supramolecules for high-efficiency organic light-emitting diodes," Nature Communications **5**, 4769 (2014).

[11] Joan Ràfols-Ribé, Paul-Anton Will, Christian Hänisch, Marta Gonzalez-Silveira, Simone Lenk, Javier Rodríguez-Viejo, and Sebastian Reineke, "High-performance organic light-emitting

diodes comprising ultrastable glass layers," Science Advances **4**, eaar8332 (2018).
